# Effects of Iron, Copper, Zinc, and Magnesium on Chronic Widespread Pain: A Two-Sample Mendelian Randomization

**DOI:** 10.3390/jcm13195908

**Published:** 2024-10-03

**Authors:** Hyunjik Kim, Dai Sik Ko

**Affiliations:** 1 Department of General Surgery, Breast Cancer Center, Gachon University Gil Medical Center, Incheon 21565, Republic of Korea; 2 Division of Vascular Surgery, Department of General Surgery, Gachon University College of Medicine, Gil Medical Center, Incheon 21565, Republic of Korea

**Keywords:** micronutrients, pain, mendelian randomization analysis, causality

## Abstract

**Background:** Chronic widespread pain (CWP) affects approximately 10% of the adult population globally, causing significant physical and psychological distress. Micronutrients, such as iron, copper, zinc, and magnesium, are essential in various physiological functions, and their imbalances may impact pain perception and chronic pain conditions. **Methods**: This study used Mendelian randomization (MR) to investigate the causal relationships between micronutrient levels and CWP. Data were obtained from genome-wide association studies (GWASs) for iron, copper, zinc, and magnesium, and CWP data were sourced from large-scale GWASs with 461,857 European participants. Genetic variants were used as instrumental variables to infer causal relationships, minimizing confounding factors. **Results**: MR analysis revealed a significant association between higher iron levels and an increased risk of CWP (IVW, OR 1.01, 95% CI: 1.00–1.01, *p* = 0.029). This finding was supported by the weighted median and MR-Egger methods. No significant associations were found for copper, zinc, and magnesium levels. **Conclusions**: These results suggest that iron levels may influence pain perception and chronic pain conditions. Balanced iron levels are crucial for managing chronic pain. Regular monitoring and personalized treatment plans could benefit individuals with CWP. Further research is needed to understand the mechanisms linking micronutrient levels to chronic pain and to develop targeted therapeutic interventions.

## 1. Introduction

Chronic widespread pain (CWP) is a debilitating condition characterized by persistent pain that has affected multiple regions of the body for at least three months [1]. It affects approximately 10% of the adult population globally [2]. Understanding and addressing CWP is crucial due to its significant impact on quality of life, leading to physical disability, psychological distress, and reduced daily functioning [3]. Addressing CWP through an improved understanding of its underlying mechanisms is crucial in developing more effective prevention and management strategies that could reduce its personal and societal impact.

Micronutrients, including iron, copper, zinc, and magnesium, play vital roles in various physiological processes. Iron is crucial to oxygen transport and energy production, while copper is involved in iron metabolism, antioxidant defense, and neurotransmitter synthesis [4]. Zinc supports immune function, DNA synthesis, and wound healing [5]. Magnesium is essential for muscle and nerve function, blood glucose control, and bone health [6]. Given their integral roles in maintaining bodily functions, deficiencies or imbalances in these micronutrients can lead to various health issues, including those related to pain and inflammation.

Epidemiological studies have increasingly explored the relationship between micronutrient levels and pain perception [7]. A systematic review of the literature found that an inadequate intake of micronutrients such as calcium, folate, zinc, magnesium, and vitamin B6 is prevalent among patients with chronic pain conditions like rheumatoid arthritis and fibromyalgia [8]. Low magnesium levels have been linked to migraine headaches and fibromyalgia, conditions often characterized by widespread pain [9]. A meta-analysis concluded that serum zinc levels in patients with rheumatoid arthritis (RA) were significantly lower than those in healthy controls, while serum copper levels were significantly higher, suggesting that zinc and copper may play a role in RA pathogenesis [10]. These findings highlight the potential role of micronutrients in modulating pain pathways, underscoring the importance of adequate nutrient intake for pain prevention and management, though the causal relationships between these micronutrients and chronic pain remain unclear.

Building on the existing body of evidence, our study aims to investigate the association between specific micronutrients (iron, copper, zinc, magnesium) and CWP using a Mendelian randomization (MR) approach. MR analysis uses genetic instruments to demonstrate causal estimates [11]. Since an individual’s genotype is established before birth, genetically predicted exposures are minimally influenced by clinical confounders or reverse causation. This genetic randomization allows for the assessment of causal effects, necessitating a large-scale genetic dataset to account for the variation in exposure phenotypes explained by the genotype [12,13].

By understanding the genetic predispositions related to micronutrient levels and their impact on CWP, our study aims to address this gap by using genetic instruments to estimate causal effects, providing novel insights into potential therapeutic targets and preventive measures for chronic pain management, including future interventions for CWP.

## 2. Methods

### 2.1. Data Source

This study used summary data from genome-wide association studies (GWASs) for iron, copper, zinc, and magnesium obtained from the IEU OpenGWAS database (https://gwas.mrcieu.ac.uk/). The iron GWAS dataset included 23,986 samples and 2,096,457 single-nucleotide polymorphisms (SNPs), subjected to genotypic imputation and quality control. Genotyping was conducted to assess the association between imputed SNPs and iron phenotypes using an additive model of allelic effects, adjusting phenotypic standardized residuals for sex after controlling for age, principal component scores, and other covariates [14]. For copper and zinc, the GWAS summary data comprised 2603 samples with 2,543,646 and 2,543,610 SNPs, respectively. Imputation was performed using the MACH Markov Chain Haplotyping software with CEU individuals from the HapMap Phase 2 as a reference. Significant SNPs at each site were included as covariates to detect independent effects, and the results were combined in a meta-analysis [15]. The magnesium GWAS data included 64,979 samples and 9,851,867 SNPs (Table 1). Genetic association data for CWP were taken from eight large-scale GWASs with 461,857 European participants, while CWP data were sourced from a GWAS with 6914 cases and 242,929 controls in the UK Biobank [16]. We downloaded summary statistics for CWP at Zenodo (https://doi.org/10.5281/zenodo.4459546, accessed on 1 March 2024). Cases were defined based on self-reported diagnoses of fibromyalgia or pain lasting more than three months in the knee, shoulder, hip, back, or body. Participants with rheumatoid arthritis, polymyalgia rheumatica, unspecified arthritis, systemic lupus erythematosus, ankylosing spondylitis, and myopathy were excluded. GWASs for CWP were adjusted for age, sex, and population stratification through principal components.

### 2.2. Genetic Instrument Selection

**Figure 1:**
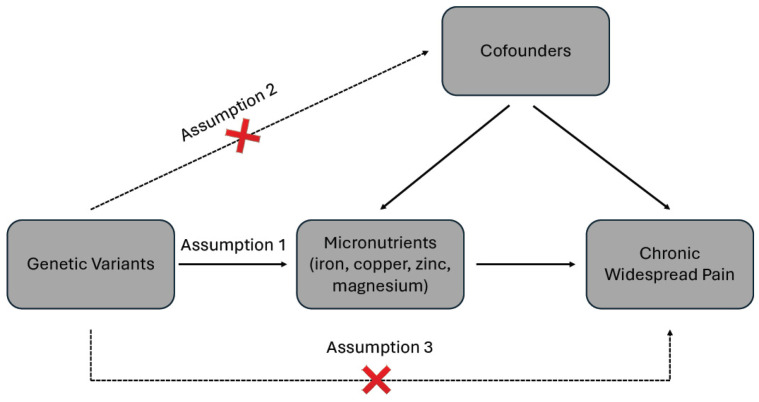
Schematic diagram of the two sample Mendelian randomizations.

Mendelian randomization (MR) analysis relies on three critical assumptions: (1) instrumental variables (IVs) must be strongly associated with the exposure; (2) IVs should not be associated with the outcome or any confounders; and (3) IVs should influence the outcome exclusively through the exposure, without any alternative pathways. These principles are depicted in Figure 1.

To ensure the validity of our MR analysis, we applied rigorous quality control measures during the selection of IVs. First, SNPs chosen as IVs had to exhibit a strong association with the exposure (*p* < 5 × 10^−6^ and F statistic > 10) [17]. The F statistic, calculated using F = R^2^(N − K − 1)/K(1 − R^2^), is employed to evaluate the strength of the correlation between IVs and exposures [18]. Here, R^2^ represents the proportion of exposure variability explained by the SNPs, N is the sample size, and K is the number of included SNPs. For individual SNPs, K equals 1 [19]. The R^2^ value was derived from the formula 2 × MAF(1 − MAF)β^2^, where β is the effect estimate of the genetic variant on the exposure, measured in standard deviation (SD) units, and MAF is the minor allele frequency [20]. An F statistic greater than 10 indicates no weak instrument bias. Second, to ensure the independence of genetic instruments, we performed LD clumping on the significant SNPs identified from the GWASs. Using PLINK software and the 1000 Genomes Project European population as a reference, we applied a clumping algorithm to group and filter SNPs. We retained only those SNPs without significant LD (r^2^ < 0.001 and clump distance > 10,000 kb). This stringent threshold was chosen to minimize the risk of bias from correlated instruments [21]. Third, SNPs significantly associated with the outcome (*p* < 5 × 10^−6^) were removed. Fourth, SNPs linked to confounding factors such as smoking, obesity, and sex were identified and excluded using the PhenoScanner database (http://www.phenoscanner.medschl.cam.ac.uk/phenoscanner, accessed on 4 March 2024) [22,23]. Additionally, palindromic SNPs with intermediate allele frequencies were omitted. If an SNP was unavailable in the outcome summary data, proxy SNPs were identified using the LDlink API with a minimum LD r^2^ of 0.8. A suitable proxy was defined as an SNP in strong LD (r^2^ ≥ 0.8) with the original SNP and present in the outcome dataset. If no suitable proxies met this criterion, then the SNP was excluded from analysis. Palindromic SNPs with intermediate allele frequencies were disregarded [24]. Supplementary Table S1 provides detailed information on the selected SNPs.

### 2.3. Statistical Analysis

To investigate the potential causal relationship between the exposure and outcome of interest, we conducted a two-sample MR analysis using the TwoSampleMR package in R version 4.2.1. Various MR methods were employed to evaluate this relationship, including the random-effects inverse-variance-weighted (IVW) method as the primary approach, with MR-Egger and weighted median methods serving as complementary analyses.

The IVW method, a classical MR statistical technique, assumes that all included SNPs are valid IVs. Wald ratios were calculated for each SNP to assess its impact on the results, and the inverse variance of the SNPs was used as the meta-analysis weight to evaluate the causal relationship between exposure and outcome [25]. The MR-Egger method offers robust estimates even when some IVs are weak instruments [26]. The weighted median method can produce reliable results even if more than 50% of the weight comes from invalid IVs, making it robust to certain violations of the MR assumptions [27]. However, compared to the IVW method, the statistical power of the MR-Egger and weighted median methods is lower. Therefore, primary conclusions about the causal relationship were derived mainly from the IVW method. Odds ratios (ORs) with 95% confidence intervals (95% CIs) were used to quantify the association between exposures and outcomes, and scatter plots were generated to visualize the direction and magnitude of these effects.

To ensure the robustness of the MR analysis results, a series of tests were conducted. Cochran’s Q statistic was used in MR-IVW and MR-Egger to detect heterogeneity, with a *p*-value > 0.05 indicating no significant heterogeneity [28]. The intercept test of the MR-Egger was used to assess horizontal pleiotropy, with a *p*-value > 0.05 suggesting no horizontal pleiotropy [29]. Additionally, a “Leave-one-out” analysis was performed to determine whether any single SNP disproportionately influenced the genetic causality assessment between exposure and outcome [30].

## 3. Results

### 3.1. Genetic Instrument Selection

We identified SNPs strongly associated with iron, copper, zinc, and magnesium exposure and subsequently removed those in LD. In total, we identified 11 SNPs associated with iron, 6 with associated copper, 8 associated with zinc, and 19 associated with magnesium. Each SNP is identified by a unique reference SNP cluster ID, commonly referred to as an “rsID” (where “rs” stands for Reference SNP). This standardized naming convention is widely used by researchers and genetic databases to uniquely identify-specific SNPs.

Among the 11 iron-associated SNPs, 1 palindromic SNP (rs7209063) was identified, and 4 SNPs (rs604302, rs7172337, rs13038647, and rs855791) were removed due to the absence of suitable proxies. For copper, 3 SNPs (rs1175550, rs10014072, and rs12582659) were removed, and no palindromic SNPs were found. In the case of zinc, one SNP (rs10931753) was identified as palindromic, but none were removed during harmonization. For magnesium, 6 SNPs (rs140205161, rs144862520, rs1247081, rs147150587, rs146963873, and rs111419911) were removed without identifying any palindromic SNPs. Finally, 6 iron-associated SNPs, 3 copper-associated SNPs, 7 zinc-associated SNPs, and 13 magnesium-associated SNPs were included in the MR analysis.

### 3.2. MR Analysis

**Figure 2:**
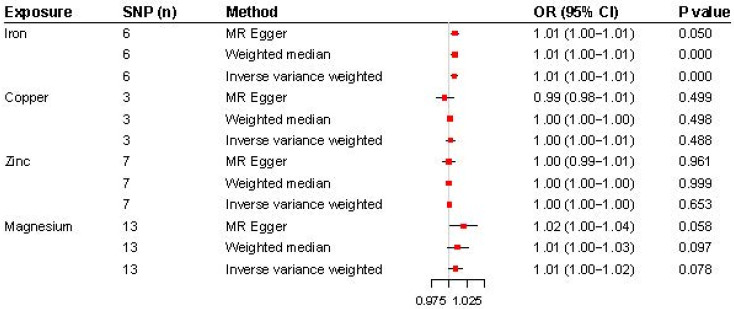
Forest plot for the MR analysis (inverse-variance-weighted) of the causal effect of micronutrients (iron, copper, zinc, and magnesium) on chronic widespread pain. OR, odds ratio; SNP, single-nucleotide polymorphism.

**Figure 3:**
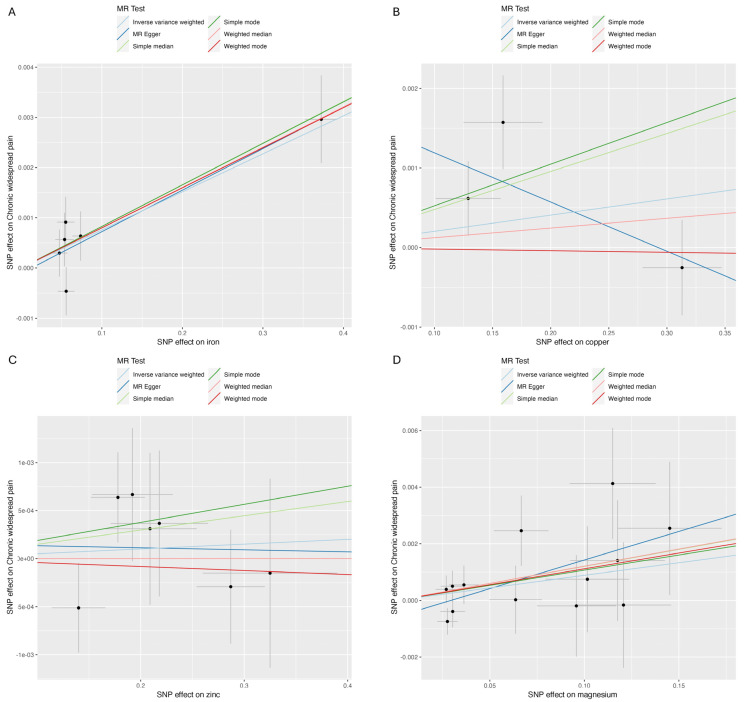
Scatter plot. (**A**) Iron and chronic widespread pain; (**B**) copper and chronic widespread pain; (**C**) zinc and chronic widespread pain; (**D**) magnesium and chronic widespread pain.

The analysis results are illustrated in Figure 2 and Figure 3. Higher genetically predicted iron levels were significantly associated with an increased risk of CWP (IVW, OR 1.01, 95% CI: 1.00–1.01, *p* < 0.001). The weighted median method (OR 1.01, 95% CI: 1.00–1.01, *p* < 0.001) and MR-Egger method (OR 1.01, 95% CI: 1.00–1.01, *p* = 0.050) supported this significant association. On the other hand, the associations between copper, zinc, and magnesium levels and CWP were not statistically significant: copper (IVW, OR 1.00, 95% CI: 1.00–1.01, *p* = 0.488), zinc (IVW, OR 1.00, 95% CI: 1.00–1.00, *p* = 0.653), and magnesium (IVW, OR 1.01, 95% CI: 1.00–1.02, *p* = 0.078). The MR-Egger and weighted median analyses also showed that copper, zinc, and magnesium were not genetically linked to CWP development.

**Figure 4:**
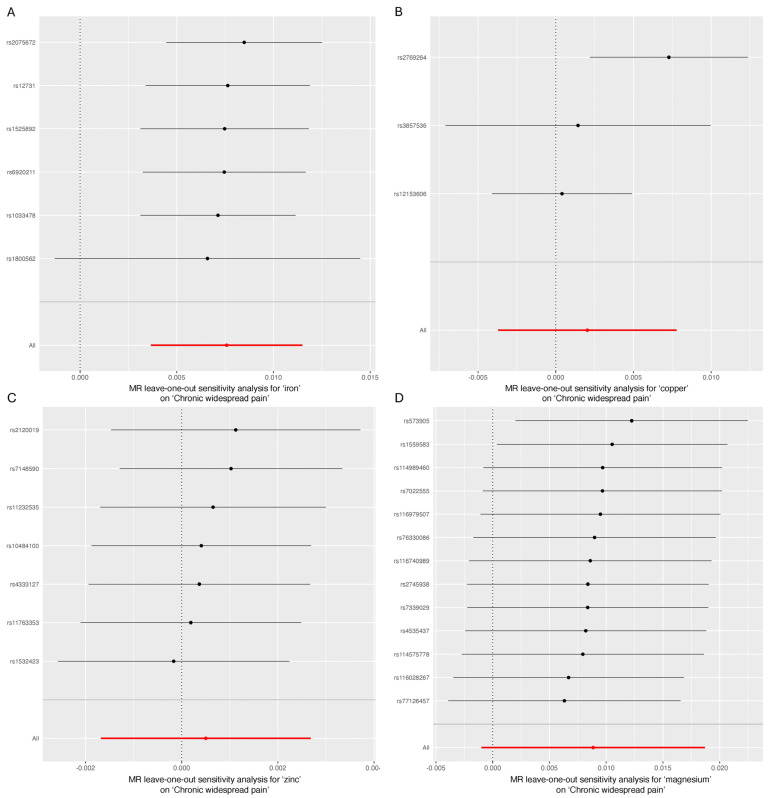
“Leave-one-out” analysis. (**A**) Iron and chronic widespread pain; (**B**) copper and chronic widespread pain; (**C**) zinc and chronic widespread pain; (**D**) magnesium and chronic widespread pain.

As shown in Table 2, Cochran’s Q statistic for the MR-IVW method indicated no evidence of heterogeneity, except in the case of copper (*p* = 0.026). The MR-Egger intercept test found no evidence of horizontal pleiotropy for iron, copper, zinc, or magnesium in relation to CWP (*p* > 0.05). Lastly, the “Leave-one-out” analysis confirmed that no single SNP significantly influenced the genetic causality assessment between these micronutrients and CWP (Figure 4).

## 4. Discussion

In this study, we used MR to explore the causal relationships between micronutrient levels (iron, copper, zinc, and magnesium) and CWP. Our MR analysis demonstrated a significant association between higher iron levels and an increased risk of CWP, with consistent results across different MR methods. No significant associations were found for copper, zinc, and magnesium.

The relationship between iron and pain has been investigated in various studies, indicating that iron levels may influence pain perception and chronic pain conditions. Low iron levels are linked to increased pain sensitivity and conditions such as restless leg syndrome and fibromyalgia [31]. Restless leg syndrome, characterized by uncomfortable leg sensations and an urge to move, is associated with iron deficiency due to its role in dopamine synthesis, critical in motor function and pain modulation [32]. Similarly, fibromyalgia, marked by widespread musculoskeletal pain, is linked to low iron levels, with studies showing reduced serum ferritin levels in individuals with fibromyalgia [33]. A study found that iron deficiency anemia increases the risk of fibromyalgia, with an adjusted hazard ratio (HR) of 1.19, while interventions like iron supplements and blood transfusions significantly reduce this risk (adjusted HR: 0.73) [34]. There is growing evidence that iron deficiency during early life significantly impacts the development of neuronal networks in the central nervous system. Studies have shown that early-life iron deficiency can lead to lasting alterations in monoamine regulation, myelination, dendritic branching, and energy metabolism [35]. Notably, these changes often persist beyond the period of acute iron deficiency and may not be fully reversed by iron supplementation later in life.

Conversely, elevated iron levels are associated with conditions like hemochromatosis, which can lead to joint pain and arthritis [36,37]. Hemochromatosis, a genetic disorder characterized by excessive iron accumulation, can cause joint pain due to oxidative damage and inflammation [38,39]. Even without hemochromatosis, higher iron levels can be linked to increased pain sensitivity and musculoskeletal pain [40,41]. Iron deposition contributes to pain through several biological processes. Iron-catalyzed oxidative reactions produce highly reactive hydroxyl radicals, damaging cellular components and triggering inflammatory pathways, leading to pain and discomfort [42]. Iron-induced oxidative stress can cause synovitis and neuroinflammation, potentially disrupting neural signaling and increasing pain sensitivity [43,44].

The findings of this study have important clinical implications for the management of CWP. Careful monitoring and management of iron levels could be a valuable component in the treatment of CWP. For patients presenting with chronic pain, assessing serum ferritin and other iron biomarkers could help identify those who might benefit from iron modulation therapies. For patients with elevated iron levels, interventions such as phlebotomy or the use of iron chelators could be considered to prevent or alleviate pain symptoms associated with iron overload. This personalized approach to managing iron levels underscores the necessity for regular monitoring and individualized treatment plans in chronic pain management. Additionally, these findings encourage the incorporation of nutritional assessments into the standard care for patients with CWP, promoting a holistic approach that addresses both nutritional deficiencies and excesses. Further clinical trials are warranted to establish specific guidelines and protocols for integrating iron management into the therapeutic regimen for chronic pain conditions.

While our study found no significant causal associations between copper, zinc, or magnesium and CWP, these null findings remain valuable in the context of pain research. Previous studies, primarily epidemiological, have suggested potential roles for copper in rheumatoid arthritis and magnesium in fibromyalgia [9,10]. However, the limitations of epidemiological studies, such as confounding factors and reverse causation, often hinder their ability to establish clear causal relationships. In contrast, our study employed an MR approach to infer causality, which may explain why we did not observe the same associations. The absence of causal links in our study also emphasizes the complexity of micronutrient interactions in pain pathways and underscores the need for further research to clarify the distinct roles that these nutrients may play in different chronic pain conditions.

Future research should focus on further elucidating the mechanisms underlying the relationship between iron levels and chronic pain. Longitudinal studies examining changes in iron levels over time and their impact on pain outcomes could provide valuable insights. Additionally, interventional studies assessing the effects of both iron supplementation and chelation therapy in individuals with chronic pain conditions could help determine the therapeutic potential of managing iron levels. Furthermore, exploring the interactions between iron and other micronutrients, as well as their combined effects on pain, could enhance our understanding of nutritional influences on chronic pain [45]. Our study provides a novel contribution by being one of the first to apply an MR approach to assessing the causal effects of micronutrients on CWP, moving beyond observational data that often fail to establish causality. This helps address the gap left by prior epidemiological studies, which are limited by confounding factors and reverse causation, by offering stronger evidence for iron’s direct role in CWP pathogenesis, while challenging the previously suggested associations of other micronutrients such as copper, zinc, and magnesium.

This study has several limitations that should be acknowledged. First, the MR approach assumes that the selected genetic instruments are not influenced by confounding factors and they may affect the outcome solely through the exposure. While we applied stringent quality control measures, the possibility of residual confounding cannot be entirely excluded. Second, the study population was predominantly of European ancestry, which may limit the generalizability of the findings to other populations. Finally, the observational nature of the GWAS data used in this study means that causal inferences should be interpreted with caution, and further experimental studies are needed to confirm our findings [46].

## 5. Conclusions

Our study provides significant insights into the association between higher iron levels and an increased risk of CWP, highlighting the need for balanced iron levels in pain management. Further research is essential in deepening our understanding of these associations. Specifically, interventional trials should be conducted to assess the effectiveness of iron modulation therapies, such as iron supplementation in iron-deficient individuals and iron chelation in those with iron overload, in reducing pain symptoms and improving the quality of life in CWP patients. By addressing these research areas, we can develop more effective, targeted therapeutic strategies for managing chronic pain conditions.

## Figures and Tables

**Table 1:** The exposure data used in this study.

	GWAS ID	Trait	Sample size	Population
exposure	ieu-a-1049	Iron	23,986	European
exposure	ieu-a-1073	Copper	2603	European
exposure	ieu-a-1079	Zinc	2603	European
exposure	ukb-b-7372	Magnesium	64,979	European

**Table 2:** Sensitivity analysis of Mendelian study.

Exposure	IVW Heterogeneity Test		MR-Egger Pleiotropy Test		
	Q	*p* Value	Intercept	SE	*p* Value
Iron	4.936	0.552	−0.0002	0.0003	0.591
Copper	7.289	0.026	0.002	0.001	0.386
Zinc	4.427	0.619	0.0002	0.0009	0.866
Magnesium	12.146	0.434	−0.0006	0.0004	0.192

IWV, inverse-variance-weighted; MR, Mendelian randomization; SE, standard error.

## Data Availability

The datasets used in this study are publicly available, and the sources have been cited and referenced accordingly.

## Supplementary Materials

The following supporting information can be downloaded at: https://www.mdpi.com/article/10.3390/jcm13195908/s1, Table S1: Characteristics of the selected SNPs for exposures (iron, copper, zinc, magnesium).
