# Supplementary material for: Effects of Iron, Copper, Zinc, and Magnesium on Chronic Widespread Pain: A Two-Sample Mendelian Randomization
Source: J Clin Med. 2024 Oct 3;13(19):5908. doi: 10.3390/jcm13195908 (PMC11478258; doi:10.3390/jcm13195908)
Supplement: Supplementary file 1 [file jcm-13-05908-s001.zip › jcm-3111814-supplementary.pdf]

Supplementary Table S1. Characteristics of the selected SNPs for exposures (iron, copper, zinc, magnesium)

Exposure	SNP	CHR	Position (GRCh38)	Effect allele	Other allele	Beta	SE	*P*-Value	R^2	F
Iron	rs12731	2	239087912	A	G	0.0473	0.01	2.52E-06	0.000932	22.37103
	rs1525892	3	133484712	A	G	0.0736	0.0104	1.65E-12	0.002084	50.07866
	rs6920211	6	135431318	C	T	0.0537	0.0115	3.14E-06	0.000908	21.80302
	rs1800562	6	26093141	A	G	0.3724	0.02	3.96E-77	0.014248	346.6755
	rs1033478	6	56088216	C	T	-0.0551	0.0108	3.63E-07	0.001084	26.02672
	rs2075672	7	100240296	G	A	0.0557	0.0103	5.95E-08	0.001218	29.24151
	rs604302	8	37004569	C	T	-0.0577	0.0124	3.07E-06	0.000902	21.6507
	rs7172337	15	61767743	C	T	0.0523	0.0111	2.63E-06	0.000925	22.19838
	rs7209063	17	1892031	G	C	-0.0483	0.0104	3.61E-06	0.000898	21.56708
	rs13038647	20	48974248	C	T	0.0463	0.01	3.80E-06	0.000893	21.43511
	rs855791	22	37462936	G	A	0.1868	0.0101	4.31E-77	0.014061	342.0383
Copper	rs2769264	1	151344741	G	T	0.313	0.034	2.63E-20	0.031531	84.68315
	rs1175550	1	3691528	G	A	0.198	0.032	5.03E-10	0.014495	38.25574

	rs10014072	4	113948790	G	A	-0.164	0.034	1.13E-06	0.008859	23.24856
	rs12153606	5	84583769	T	G	-0.159	0.034	2.50E-06	0.008332	21.85257
	rs3857536	6	66929048	T	C	-0.129	0.028	4.08E-06	0.008088	21.20946
	rs12582659	12	76064528	C	T	1.262	0.27	2.86E-06	0.008323	21.83018
Zinc	rs10931753	2	154568757	C	G	-0.129	0.028	4.94E-06	0.008088	21.20946
	rs4333127	4	5930033	A	G	0.218	0.047	3.00E-06	0.008197	21.49728
	rs11763353	7	15630871	G	A	-0.192	0.039	6.90E-07	0.009225	24.21806
	rs1532423	8	86268313	G	A	-0.178	0.026	6.40E-12	0.017688	46.83381
	rs11232535	11	80928809	C	T	0.325	0.065	6.73E-07	0.009513	24.98079
	rs7148590	14	65473196	A	G	-0.14	0.026	1.37E-07	0.011016	28.97181
	rs10484100	14	86817096	G	A	-0.209	0.045	3.30E-06	0.008219	21.55429
	rs2120019	15	75334184	C	T	-0.287	0.033	1.55E-18	0.028237	75.57917
Magnesium	rs2745938	1	208139949	T	G	0.0269243	0.00561835	1.60E-06	0.000353	22.96455
	rs116740989	2	104993244	A	C	0.117547	0.0250756	2.80E-06	0.000338	21.9739
	rs114575778	4	79596378	A	G	-0.145164	0.0274331	1.20E-07	0.000431	27.99979
	rs140205161	4	88358683	G	A	0.162252	0.0352098	4.10E-06	0.000327	21.23438

	rs116028267	5	179052804	G	C	-<br>0.0666131	0.014422	3.90E-06	0.000328	21.33315
	rs4535437	5	79242663	G	A	0.0302082	0.00623112	1.20E-06	0.000362	23.50196
	rs114989460	6	46322293	C	T	0.0957603	0.0208955	4.60E-06	0.000323	21.00159
	rs144862520	7	153872931	T	C	-<br>0.0703248	0.0153669	4.70E-06	0.000322	20.94262
	rs77126457	8	136152474	A	G	0.114984	0.0227843	4.50E-07	0.000392	25.46772
	rs76330086	9	110307810	T	C	-0.101588	0.0221236	4.40E-06	0.000324	21.08433
	rs7022555	9	137753121	T	C	-0.120695	0.025269	1.80E-06	0.000351	22.81335
	rs1247081	10	30048948	T	G	0.0256696	0.00536614	1.70E-06	0.000352	22.88236
	rs116979507	11	90779474	T	C	0.063628	0.0138895	4.60E-06	0.000323	20.98505
	rs573905	11	118572267	G	A	0.0275402	0.00538992	3.20E-07	0.000402	26.10695
	rs147150587	12	28448965	A	G	0.128307	0.0276315	3.40E-06	0.000332	21.56147
	rs7339029	13	100899519	T	G	0.0362754	0.00792269	4.70E-06	0.000323	20.96359
	rs146963873	16	70945259	A	T	-0.16778	0.0329752	3.60E-07	0.000398	25.88762
	rs1559583	18	70714102	C	T	-	0.00654176	3.50E-06	0.000331	21.50672

						0.0303381				
	rs111419911	20	38900761	G	A	- 0.0292815	0.00587917	6.30E-07	0.000382	24.80511

CHR, chromosome; SE, standard error; SNP, single nucleotide polymorphism
